# Supplementary material for: Development of Certified Reference Materials for the Determination of Apparent Amylose Content in Rice
Source: Molecules. 2022 Jul 21;27(14):4647. doi: 10.3390/molecules27144647 (PMC9322866; doi:10.3390/molecules27144647)
Supplement: Supplementary file 1 [file molecules-27-04647-s001.zip › molecules-1808897-supplementary.pdf]

Supplementary Materials

Development of certified reference materials for the determination of apparent amylose content in rice

Supplementary Table S1. Moisture content (%) of the four rice reference materials for five replicates

Samples	1	2	3	4	5	Mean ± SD
RM01	10.56	10.44	10.45	10.54	10.44	10.49 ± 0.06
RM02	11.30	11.40	11.36	11.37	11.34	11.35 ± 0.04
RM03	10.92	10.88	10.87	10.85	10.91	10.89 ± 0.03
RM04	9.82	9.71	9.81	9.77	9.77	9.78 ± 0.04

Supplementary Table S2. The iodine binding capacity of the stock potato amylose

No.	V (mL)	m (mg)	ω_{m} (%)	x (%)	m ± sd
1	1.139	5.0	10.18	19.30	
2	1.140	5.0	10.18	19.31	19.28 ± 0.04
3	1.136	5.0	10.18	19.25	

The iodine binding capacity of the stock potato amylose was shown as mean (m) ± standard deviation (sd) (n =3), and calculated as follows:

$$x = \frac{0.7610}{m(1-\omega_m)} \times V \times 100$$

where

x is the iodine binding capacity;

m is the mass of potato amylose;

ω_{m} is the moisture content of the potato amylose;

V is the volume of stock potassium iodate solution needed to titrate the amylose solution;

0.7610 is a factor to allow for 1 mL of stock potassium iodate solution being equivalent to 0.7610 mg of iodine.

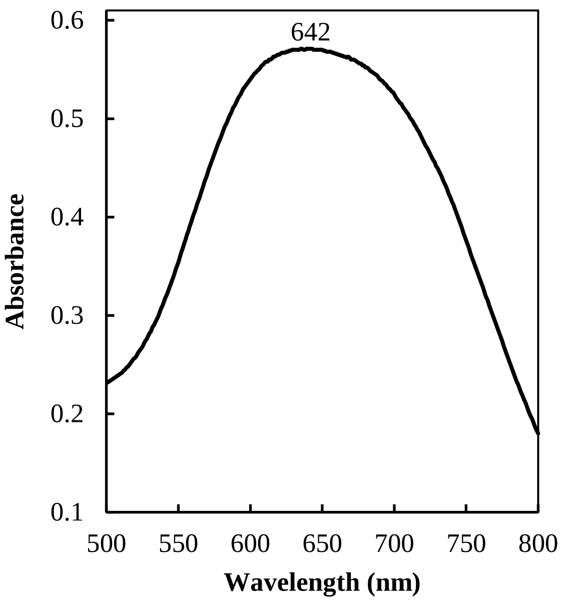

Supplementary Figure S1. The maximum absorbance of the iodine-starch complex of potato amylose standard
